# Successive Inoculations of Pigs with Porcine Reproductive and Respiratory Syndrome Virus 1 (PRRSV-1) and Swine H1N2 Influenza Virus Suggest a Mutual Interference between the Two Viral Infections

**DOI:** 10.3390/v13112169

**Published:** 2021-10-27

**Authors:** Juliette Bougon, Céline Deblanc, Patricia Renson, Stéphane Quéguiner, Stéphane Gorin, Sophie Mahé, Mireille Le Dimna, Nicolas Barbier, Frédéric Paboeuf, Gaëlle Simon, Olivier Bourry

**Affiliations:** 1 French Agency for Food, Environmental and Occupational Health and Safety (ANSES), Ploufragan-Plouzané-Niort Laboratory, Swine Virology Immunology Unit, BP53, 22440 Ploufragan, France; juliette.bougon@anses.fr (J.B.); celine.deblanc@anses.fr (C.D.); patricia.renson@anses.fr (P.R.); stephane.queguiner@anses.fr (S.Q.); stephane.gorin@anses.fr (S.G.); sophie.mahe@anses.fr (S.M.); mireille.ledimna@anses.fr (M.L.D.); nicolas.barbier@anses.fr (N.B.); olivier.bourry@anses.fr (O.B.); 2 University of Rennes 1, Cité Internationale, 1 Place Paul Ricoeur, CS 54417, 35044 Rennes, France; 3 ANSES, Ploufragan-Plouzané-Niort Laboratory, SPF Pig Production and Experimentation Unit, BP53, 22440 Ploufragan, France; frederic.paboeuf@anses.fr

**Keywords:** swine influenza A virus, Betaarterivirus suid 1, porcine respiratory disease complex, virus-virus interaction, viral interference, innate and adaptive immune responses, inflammatory response, interferon, super-infection

## Abstract

Porcine reproductive and respiratory syndrome virus (PRRSV) and swine influenza A virus (swIAV) are major pathogens of the porcine respiratory disease complex, but little is known on their interaction in super-infected pigs. In this study, we investigated clinical, virological and immunological outcomes of successive infections with PRRSV-1 and H1N2 swIAV. Twenty-four specific pathogen-free piglets were distributed into four groups and inoculated either with PRRSV at study day (SD) 0, or with swIAV at SD8, or with PRRSV and swIAV one week apart at SD0 and SD8, respectively, or mock-inoculated. In PRRSV/swIAV group, the clinical signs usually observed after swIAV infection were attenuated while higher levels of anti-swIAV antibodies were measured in lungs. Concurrently, PRRSV multiplication in lungs was significantly affected by swIAV infection, whereas the cell-mediated immune response specific to PRRSV was detected earlier in blood, as compared to PRRSV group. Moreover, levels of interferon (IFN)-α measured from SD9 in the blood of super-infected pigs were lower than those measured in the swIAV group, but higher than in the PRRSV group at the same time. Correlation analyses suggested an important role of IFN-α in the two-way interference highlighted between both viral infections.

## 1. Introduction

Porcine respiratory disease complex (PRDC) is a major issue for pig producers worldwide, because of economic impacts in swine herds and public health concerns due to the excretion of zoonotic pathogens and antibiotics treatments. PRDC is a multifactorial disease due to simultaneous or sequential viral and/or bacterial infections which could induce severe respiratory troubles leading to poor animal growth in the case of additive or synergic outcomes [1]. The PRDC manifestation could depend on environmental factors, breeding practices, farm management and host-dependent factors such as age, genetics or immune status. However, PRDC severity could also closely depend on the interactions between pathogens themselves. Previous studies showed that respiratory viral infections can promote an ideal environment for bacterial super-infections through the destruction of the epithelial barrier, the over-expression of receptors involved in bacterial adhesion to the cells and the alteration of host immune system [1,2]. Conversely, a pre-existing chronic pulmonary bacterial infection, such as *Mycoplasma hyopneumoniae* (Mhp) infection, could potentiate the outcome of a subsequent viral infection by inducing production of pro-inflammatory cytokines and recruitment of immune cells [2,3]. Unfortunately, only few studies have explored the virus/virus interactions that may occur in pig respiratory tract. Among the viruses involved in PRDC, porcine reproductive and respiratory syndrome virus (PRRSV) and swine influenza A virus (swIAV) are among the most important primary agents [1]. These viruses are both highly prevalent and enzootic in pig populations from several European countries [4,5]. PRRSV is an enveloped virus with a positive-sense single-stranded RNA genome that belongs to the genus Betaarterivirus of the *Arteriviridae* family. There are two PRRSV species, Betaarterivirus suid 1 (PRRSV-1) and Betaarterivirus suid 2 (PRRSV-2), mostly widespread in Europe and North America/Asia, respectively [6,7]. This virus primarily infects alveolar macrophages [8], is responsible for long-lasting infections [9,10], and is considered a causative agent of reproductive troubles, respiratory disorders and growth retardation [11]. Most PRRSV strains interfere with innate antiviral immune responses by blocking interferon-alpha (IFN-α) signaling and promoting the secretion of immunosuppressive cytokines, such as interleukin-10 (IL-10) [12]. swIAV is an enveloped virus with a negative-sense, single-stranded and segmented RNA genome that belongs to the genus Influenzavirus A of the *Orthomyxoviridae* family [13]. There are three swIAV subtypes, i.e., H1N1, H1N2 and H3N2, which circulate simultaneously in areas of high pig density, with multiple region-specific genetic lineages within each subtype [14,15]. swIAV target cells are epithelial cells of the upper and lower respiratory tracts [13]. swIAV infection is responsible for acute respiratory disease, characterized by fever, cough, sneezing, apathy and dyspnea, associated with growth retardation [16]. Unlike PRRSV, swIAV is known to be a high inducer of type-I IFN and inflammatory responses [17]. In production areas where PRRSV and swIAV are widespread, there is a high risk that pig farms are infected by both viruses at the same time [18,19]. However, very few studies have analyzed the impact of a PRRSV/swIAV super-infection in pigs. They have mainly focused on clinical and virological parameters, but with inconsistent results; some studies have reported exacerbations of lung lesions [20,21,22], whereas others showed no difference in clinical signs as compared to single-infected animals [21,23,24]. Furthermore, the host immune responses have been studied only to a very limited extent at the systemic or pulmonary levels. Considering the lacks and apparent contradictions of these previous studies, new investigations were necessary to better characterize the effect one virus infection may have on the other, as well as the impact of dual PRRSV/swIAV infection on the host immune responses.

In Brittany, France, epidemiological investigations in farrow-to-finish farms showed that seropositive status towards infection with swIAV from the “European human-like reassortant swine H1N2” (H1_hu_N2) lineage, but not the “European avian-like swine H1N1” (H1_av_N1) lineage, was significantly associated with seropositive status towards PRRSV-1 infection [25]. In this context, we aimed, in an experimental study, (i) to investigate the clinical and immuno-virological impact of a PRRSV-1 infection on a subsequent H1_hu_N2 swIAV infection, and (ii) to explore the effect of H1_hu_N2 swIAV infection on the course of ongoing PRRSV-1 infection. Knowing that the impact of PRRSV-1 single-infection in lungs of specific-pathogen-free (SPF) pigs is a maximum of one week post-inoculation, with both the highest PRRSV-1 genomic load and the lowest alveolar macrophage count [10], we delayed swIAV inoculation by eight days after that of PRRSV-1. Thus, pigs were inoculated either with PRRSV at study day (SD) 0, or with swIAV at SD8, or with PRRSV and swIAV at SD0 and SD8, respectively, or mock-inoculated. Monitoring of clinical outcomes and viral multiplications, as well as studies of innate and adaptive immune responses specific to each virus at both the systemic and pulmonary levels, were implemented for four weeks post-inoculation with PRRSV.

## 2. Materials and Methods

### 2.1. Virus Strains and Titrations

The PRRSV-1 subtype 1 Finistere strain (PRRS-FR-2005-29-24-1) (GenBank accession No. KY366411) was isolated in 2005 from a sow with reproductive failures in a herd located in Brittany, France. Infectious particles of PRRSV-1 were propagated and titrated on primary porcine alveolar macrophages (PAMs) collected from SPF piglets for three and four passages for in vivo inoculations and enzyme-linked immunospot (ELISPOT) assays, respectively. Serial 10-fold dilutions of samples were performed in Roswell Park Memorial Institute medium (RPMI, Thermo Fisher Scientific, Waltham, MA, USA) supplemented with 4% of penicillin–streptomycin, 10% of fetal bovine serum, 1% L-Glutamin and incubated at 37 °C, 5% CO_2_. The cytopathic effects were observed after five to seven days. Virus titers were calculated using the Karber method [26].

The swIAV strain A/Sw/France/Ille et Vilaine-0415/2011 of H1_hu_N2 subtype (GenBank accession No. KR699787-94) was isolated from a pig with acute respiratory disease during an outbreak in a pig herd located in Brittany, France. Infectious particles of swIAV were propagated and titrated on Madin–Darby canine kidney (MDCK) cells for five passages. Serial 10-fold dilutions of samples were performed in minimal essential medium (MEM, Thermo Fisher Scientific, Waltham, MA, USA) supplemented with 2% of penicillin–streptomycin and 2 µg/mL Trypsine TPCK (Worthington, Biochemical Corporation, Lakewood, NJ, USA) and incubated at 37 °C, 5% CO_2_. The cytopathic effects were observed after three to four days. Virus titers were calculated using the Reed and Muench method [27].

### 2.2. Animal Experiment Design

Twenty-four 8-week-old SPF piglets were used in this study. They were obtained from the air-filtrated pig herd of the French Agency for Food, Environmental, and Occupational Health and Safety (ANSES, Ploufragan, France). The piglets were randomly distributed into four groups of six animals and housed into four independent rooms (Table 1) in our biosecurity level 3 animal facilities. PRRSV-1 Finistere strain (5 × 10^5^ TCID_50_ in a volume of 5 mL per pig) was inoculated intranasally at SD0 to the pigs from PRRSV and PRRSV/swIAV groups. swIAV H1_hu_N2 strain (10^6^ TCID_50_ in a volume of 5 mL per pig) was inoculated intratracheally at SD8 to the pigs from swIAV and PRRSV/swIAV groups. For mock-inoculations and the Control group, 5 mL of MEM was inoculated, intranasally at SD0 and intratracheally at SD8. 

The experiment was performed in the ANSES facilities, which have an agreement for animal experimentation, delivered by the Direction Départementale de la Protection des Populations des Côtes d’Armor (ANSES registration number C-22-745-1). The animal experiment was authorized by the French Ministry for Research (authorization no. APAFIS#19874-2019032011429342 v3) and approved by the National Committee for Ethics in Animal Experimentation ANSES/ENVA/UPEC n°16 (approval no. 19-023#19874).

### 2.3. Clinical Monitoring, Sampling and Necropsy

Clinical signs (rectal temperature, cough and sneezing) were daily recorded, as previously described [28]. Hyperthermia was assigned to rectal temperature higher than 40 °C. Cough and sneezing were counted for 15 min in each room. Individual weighing and food consumption in each pen were measured weekly. 

Blood samples and broncho-alveolar lavages (BAL) were collected at SD-2, 7, 9, 12, 15, 21, 26 in all groups. Serum samples were collected from coagulated blood samples (3000× *g* for 5 min centrifugation) and frozen at −20 °C for antibody measurements and cytokine analyses, and −80 °C for viral titration and RT-qPCR analyses. Peripheral blood mononuclear cells (PBMCs) were isolated from heparinized blood by Ficoll-density gradient centrifugation with LeucoSep tubes (Greiner Bio One, Les Ulis, France) and frozen in liquid nitrogen using dimethyl sulfoxide (DMSO)-containing cryopreservation medium (Sigma, Saint-Quentin-Fallavier, France) until analyses to monitor cellular immune responses. BAL collection was performed under general anesthesia following the intramuscular injection of 10 mg/kg Zoletil (Virbac, Carros, France) and were obtained by infusing 2 × 20 mL of sterile phosphate-buffered saline (PBS) using a tracheal suction probe (Vygon, Ecouen, France), where size and diameter depended on the age and weight of animals. BAL fluid (BALF) was collected after centrifugation of the BAL at 400× *g* for 8 min at 4 °C and frozen at −20 °C until antibody and cytokine analyses, and −80 °C for viral titration and RT-qPCR analyses. BAL cells (BALCs) were isolated after BAL centrifugation and frozen in the same way as PBMCs until flow cytometry analyses.

Nasal swabs were sampled daily from SD8 for one week, then once a week, in the PRRSV/swIAV and swIAV groups. In the PRRSV group, nasal swabs were only collected at the times of blood and BAL collections. Nasal swabs were suspended in Virocult (MW915 sent, Virocult^®^, Corsham, UK) and supernatants were frozen at −80 °C until RT-qPCR and viral titration analyses. 

The pigs were euthanized using anesthesia with 10 mg/kg of Zoletil followed by bleeding and necropsied between SD27 and SD29 (Table 1). Post-mortem examination and scoring of pneumonia lesions was performed as previously described [29]. 

### 2.4. Quantifications of PRRSV and swIAV Genomic Loads

The PRRSV genome was quantified in sera, BALF and nasal swab supernatants. Viral RNA was extracted by the KingFisherTM Flex automated extraction robot (Thermo Fisher Scientific, Waltham, MA, USA) using the Maxwell^®^ HT 96 gDNA Blood kit (Promega, Lyon, France). Detection and quantification of PRRSV Finistere strain was assessed by duplex reverse transcription–quantitative real-time PCR (RT-qPCR) using the SuperScript III Platinum one-step RT-qPCR kit (Life Technologie, Carlsbad, CA, USA) with primers and probes specific to ORF5 and β-actin as previously described [10]. RT-qPCR was performed on a Chromo4 real-time PCR device (Biorad, Hercules, CA, USA). The RT-qPCR conditions were: 50 °C for 30 min, 94 °C for 2 min, 45 cycles each of 94 °C for 15 s and 60 °C for 30 s. Virus genome quantification was obtained by using serial dilutions of PRRSV Finistere strain with a known infectious titer in BALF or serum collected in SPF pigs. The PRRSV genome amount was expressed as equivalent TCID_50_/mL, as previously reported [30,31].

The swIAV genome was quantified in nasal swab supernatants and BALF. swIAV RNA was extracted using the same protocol as for PRRSV RNA. Detection and quantification of the swIAV M gene was assessed by duplex M/β-actin RT-qPCR using a Go Script RT mix for 1-Step RT-qPCR (Promega, Madison, WI, USA), as previously described [32]. RT-qPCR was performed on an MX 3005P real-time PCR device (Stratagene, CA, USA). The RT-qPCR conditions were: 45 °C for 30 min, 95 °C for 2 min, 40 cycles each of 95 °C for 15 s, 60 °C for 1 min. Virus genome quantification was obtained by using serial dilutions of standardized M and β-actin mRNA. The swIAV genome amount was expressed as copy numbers of M gene/10^6^ copies of β-actin gene.

### 2.5. Haptoglobin and Cytokine Measurements

Haptoglobin concentrations in serum were measured using a Phase Haptoglobin colorimetric Assay kit (Tridelta, Maynooth, Ireland). Porcine IFN-α was quantified in serum and BALF by an in-house enzyme-linked immunosorbent assay (ELISA) [33]. Commercial ELISA kits were used to measure porcine IL-6 (Bio-Techne, Minneapolis, MN, USA), IL-4, IFN-γ and IL-10 (Invitrogen, CA, USA) in serum and/or BALF. IL-6 and IL-10 concentrations were low and close to the quantification threshold of the ELISA kits; therefore, we considered these cytokines to be induced when the optical density (OD) of the sample was higher than the basal level observed in uninfected pigs, i.e., the mean OD + 2 standard deviations calculated for the Control group.

### 2.6. Blood Cell Count and BALC Phenotyping

Total blood counts were performed with an MS9.5 hematology analyzer (Melet Schloesing Laboratoires, Osney, France) from EDTA blood samples. 

Phenotyping of macrophages and granulocytes from BAL was performed by flow cytometry analysis on frozen BALCs. BALCs were thawed, washed in PBS (400× *g* for 5 min at 4 °C), transferred to 96-well plates (0.5 × 10^6^ cells per well) and then single- or triple-stained with the following primary mouse monoclonal antibodies for 30 min at 4 °C: R-phycoerythrin (RPE)-conjugated anti-pig CD172α (clone 74-22-15), fluorescein isothiocyanate (FITC)-conjugated anti-pig CD203α (clone PM18-7), unlabeled anti-pig SWC8 (clone MIL3) (BioRad, Hercules, CA, USA and Southern Biotech, Birmingham, AL, USA) or stained with appropriate mouse isotype control, RPE or FITC-coupled IgG1 (Biorad) and unlabeled mouse IgM (Life Technologies, Carlsbad, CA, USA). The unlabeled primary antibody was detected by a secondary FITC-conjugated human anti-mouse IgM antibody (Biorad). Antibodies were used at the concentrations that were recommended by the manufacturers. A 7-Amino-Actinomycin D (7-AAD) cell viability solution was used to exclude dead cells, according to the recommendation of the manufacturer (BD Biosciences, San Jose, CA, USA). For each immunostaining, data from 30,000 events were acquired with an FC500 cytometer and analyzed with Kaluza 1.2 software (Beckman Coulter, Fullerton, CA, USA).

### 2.7. Antibody Assessment

Anti-PRRSV (protein N) immunoglobulins G (IgG) were detected with IDEXX PRRS X3 ELISA kit (IDEXX laboratories, Liebefeld, Switzerland), in serum following the manufacturer’s instructions (dilution 1:40), and in BALF using an adapted protocol (dilution 1:2). Anti-PRRSV immunoglobulins A (IgA) were detected in BALF (dilution 1:2) with the same kit as IgG with a modified protocol using goat anti-pig IgA antibody HRP conjugate (Euromedex, Souffelweyersheim, France) at a 1:3000 dilution as a conjugated antibody. 

Anti-swIAV (protein NP) IgGs were detected with the ID Screen Influenza A Nucleoprotein Swine Indirect kit (Innovative Diagnostics, Grabels, France) in serum (dilution 1:100) and BALF (dilution 1:2). Anti-swIAV IgAs were detected in BALF (dilution 1:50) with the same kit using goat anti-pig IgA antibody HRP conjugate (Euromedex, Souffelweyersheim, France) at a 1:3000 dilution as a conjugated antibody. 

For anti-PRRSV and anti-swIAV IgA assays in BALF, the negative and positive controls included in the commercial kits were replaced by in-house BALF controls, calibrated as those for anti-PRRSV or anti-swIAV IgG from each kit to calculate sample-to-positive (S/P) ratios.

### 2.8. Hemagglutination Inhibition Assay

Antibodies directed against the swIAV H1_hu_N2 hemagglutinin (HA) were titrated in sera and BALF collected at SD21 and SD26 using the hemagglutination inhibition (HI) assay following standard procedures [34]. Briefly, RDE (*Vibrio cholerae* Receptor-Destroying Enzyme) treatment and chicken erythrocyte-adsorbed were performed on samples to eliminate non-specific HA inhibitors and non-specific agglutinins. These samples were serially diluted two-fold from 1/10 to 1/2560. The challenge swIAV strain A/Sw/France/Ille et Vilaine-0415/2011 (H1_hu_N2) was used as a virus antigen. Four hemagglutinating units (HAUs) of antigen were added to each well and incubated for 35 min at room temperature. A suspension of 40 × 10^6^ chicken erythrocytes per milliliter was then added to each well, and HI titers were read after incubation for 35 min at room temperature. HI titers equal to or greater than 10 were considered to be positive.

### 2.9. Virus Neutralization Assays

PRRSV-specific neutralizing antibodies were detected in sera collected at SD26. Sera were heat-inactivated at 56 °C for 30 min. Then, they were serially diluted two-fold from 1/5 to 1/320 and 50 µL samples of each dilution were incubated in duplicate in 96-well microtiter plates with the PRRSV DV strain (GenBank accession No. MW674756), a PRRSV-1 reference strain close to the Finistere strain, at 10^1± 0.5^ TCID_50_/50 µL for 1 h at 37 °C, 5% CO_2_, with rocking agitation. A suspension of Meat Animal Research Center-145 (MARC-145) cells (0.5 × 10^5^ per well) was then added to each well, and after incubation for five to seven days at 37 °C, 5% CO_2_, the titers were determined as the reciprocal of the highest dilution of serum that prevents virus infection of the cell monolayer, as determined by the absence of cytopathic effect in half of the duplicate wells. The titers were log2-transformed in order to calculate the mean neutralizing titer of each group.

swIAV-specific neutralizing antibodies were titrated in sera and BALF collected at SD21 and SD26. Sera and BALF were previously RDE-treated and chicken erythrocytes-adsorbed to reduce non-specific reactions, as described above. Then, they were serially diluted two-fold from 1/20 to 1/20,480 and 50 µL samples of each dilution were incubated in duplicate in 96-well microtiter plates with the swIAV strain A/Sw/France/Ille et Vilaine-0415/2011 (H1_hu_N2) used as a virus antigen, at 10^1.5^TCID_50_/50 µL for 1 h at 37 °C, 5% CO_2,_ with rocking agitation. Then, the virus/sample mixtures were inoculated into adherent MDCK cells (3 × 10^4^ cells per well) for 1.5 h at 37 °C, 5% CO_2_ with rocking agitation. After two washings, samples were incubated with 100 µL of MEM supplemented with 2% of penicillin–streptomycin and 2 µg/mL trypsine TPCK for three to four days at 37 °C, 5% CO_2_. The titers were determined using the same method as for PRRSV-specific neutralizing antibodies. 

### 2.10. Quantification of IFN-γ-Secreting Cells

PRRSV and swIAV-specific IFN-γ-secreting cells (IFN-γ-SCs) were quantified by ELISPOT using a protocol adapted to frozen PBMC. ELISPOT assays were performed in triplicate. Millipore MultiScreen 96-well plates (Millipore, Burlington, MA, USA) were coated with 0.5 µg/well of purified mouse anti-pig IFN-γ antibody (clone P2G10, BD Biosciences, San Jose, CA, USA) overnight at 4 °C. For each stimulation, 4 × 10^5^ PBMCs were incubated at 37 °C, 5% CO_2_ during 42 h at a multiplicity of infection (MOI) of 0.5 for PRRSV-1 Finistere strain and an MOI of 1 for swIAV strain A/Sw/France/Ille et Vilaine-0415/2011 (H1_hu_N2). Positive control was performed by stimulating PBMCs with 10 µg/mL of phytohemagglutinin (Eurobio, Courtaboeuf, France), and negative control by stimulating PBMCs with MEM. IFN-γ was detected by adding 50 µL/well of biotinylated mouse anti-pig IFN-γ antibody (clone P2C11, BD Biosciences, San Jose, CA, USA) at 0.5 µg/mL for two hours and then 50 µL/well of streptavidin alkaline phosphatase (1:1000 dilution, Thermo Fisher Scientific, Waltham, MA, USA) for one hour at room temperature. The number of spots representing specific IFN-γ-SCs were revealed by the alkaline phosphatase conjugate substrate kit (BioRad, Hercules, CA, USA). The number of spots per well was counted with an ImmunoSpot S6 UV Analyzer (CTL, Shaker Heights, OH, USA). The number of IFN-γ-SCs was obtained by subtracting the number of non-specific spots from negative control to the number of spots obtained with PRRSV or swIAV stimulations. The results are expressed as the number of IFN-γ-SCs per 10^6^ PBMC.

### 2.11. Statistical Analysis

Data were analyzed by using non-parametric Kruskal–Wallis test with Holm’s corrected pairwise comparisons to study difference between groups. For haptoglobin measurement, a Holm’s adjusted pairwise Wilcoxon–Mann–Whitney test using paired parameters was used to assess differences between time points within each group. Principal component analyses (PCAs) were performed with 13 variables (Figure S1) when analyzing PRRSV/swIAV and PRRSV groups, and 20 variables (Figure S2) when analyzing PRRSV/swIAV and swIAV groups. All variables studied were listed according to the SD and sample matrix (Table 2). PCA graphics were produced using the FactoMineR R packages and the correlation analysis was performed with a bilateral Spearman’s non-parametric test. Correlation analyses between all variables were also performed using the Spearman rank correlation test (Figures S1 and S2). All the statistical analyses were performed using R software (version 3.1.3), and significant differences were considered when *p* < 0.05.

## 3. Results

### 3.1. PRRSV Pre-Infection Mitigated the Clinical Impact of swIAV Infection

**Figure 1:**
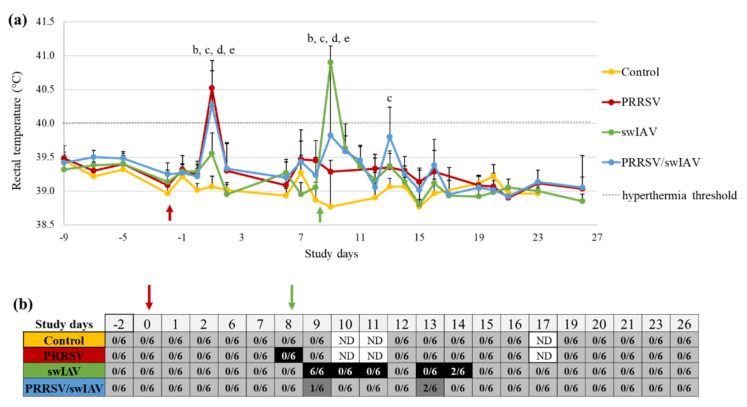
Clinical signs. (**a**) Rectal temperature. All data are reported as the mean (±standard deviation) of results obtained from pigs (*n* = 6) in the PRRSV/swIAV (blue), PRRSV (red), swIAV (green) or Control (yellow) groups. Study days: SD. SD0 (red arrow): PRRSV inoculation; SD8 (green arrow): swIAV inoculation. (**b**) Respiratory signs. *n*/6: number of pigs with rapid breathing out of the number of pigs in the group. Black box: detection of cough, sneezing in the group and rapid breathing occasionally; Dark grey box: detection of rapid breathing only; Light grey box: no respiratory signs; ND: no data; Letters indicate that significant differences (with *p* < 0.05) were obtained between PRRSV/swIAV and (a) PRRSV or (b) swIAV or (c) Control groups, (d) and (e) significant difference between PRRSV or swIAV and Control groups (respectively). Only significant differences for groups exhibiting hyperthermia are shown in this figure.

In order to compare the clinical outcomes of PRRSV/swIAV super-infection to that of PRRSV or swIAV single infections, rectal temperature and respiratory signs (cough, sneezing and breathing frequency) were followed-up daily from SD0. Animals from PRRSV and PRRSV/swIAV groups showed hyperthermia (rectal temperature > 40 °C) at SD1, with mean rectal temperatures of 40.5 ± 0.3 °C and 40.3 ± 0.7 °C, respectively, that were both significantly higher than in Control and swIAV groups (*p*-value (*p*) < 0.0001) (Figure 1a). The day after swIAV inoculation (SD9), all (6/6) animals in the swIAV group exhibited hyperthermia, but only 4/6 did in the PRRSV/swIAV group, resulting in a significant difference in mean rectal temperatures between both groups (40.9 ± 0.2 °C and 39.8 ± 1.0 °C, respectively, *p* = 0.0025). At SD13, the PRRSV/swIAV group further displayed a slight increase in mean rectal temperature, significantly higher than in the Control group (*p* = 0.0008), but under the hyperthermia threshold. No or only minor respiratory troubles were observed in the PRRSV group in the time course of the experiment (Figure 1b). Cough and sneezing were recorded in the swIAV group during the first week after swIAV inoculation, at SD9-11 and SD13-14. Moreover, 6/6 pigs exhibited rapid breathing at SD9 and 2/6 pigs at SD14. In comparison, cough and sneezing were not detected in the PRRSV/swIAV group, and only 1/6 and 2/6 pigs exhibited rapid breathing at SD9 and SD14, respectively. 

There was no difference observed between the groups in terms of growth performance, based on weighing and food consumption measurements once a week. At necropsy, three weeks after swIAV inoculation, no macroscopic lung lesions were observed in any group. Altogether, these results indicated that PRRSV pre-infection did not exacerbate, and even led to an attenuation of the influenza syndrome as usually observed after intratracheal swIAV inoculation in SPF pigs.

### 3.2. swIAV Infection Markedly Disrupted Ongoing PRRSV Multiplication in Lungs

PRRSV and swIAV multiplications were monitored in BALF, nasal swab supernatants and/or serum samples from all infected groups by RT-qPCR and virus titrations. No PRRSV or swIAV genome was detected in BALF or nasal swab supernatants sampled in the Control group. Additionally, the PRRSV genome was not detected in the blood of Control animals. 

**Figure 2:**
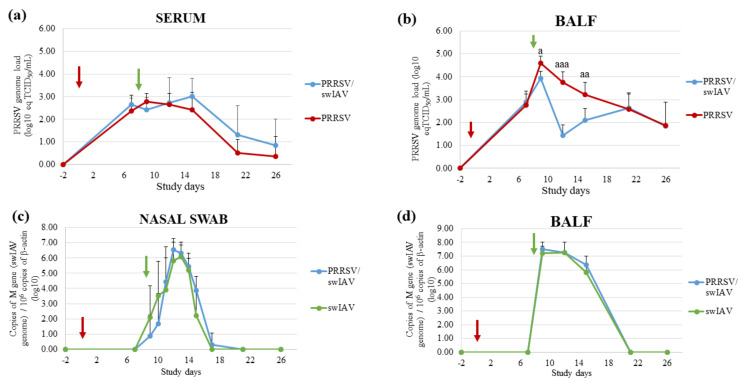
PRRSV or swIAV genomic load in sera, BALF and nasal swab supernatants from inoculated groups. PRRSV genomic loads quantified in sera (**a**) and BALF (**b**). swIAV genomic loads quantified in nasal swab supernatants (**c**) and BALF (**d**). All data are reported as the mean (±standard deviation) of results obtained from pigs (*n* = 6) in the PRRSV/swIAV group (blue), PRRSV group (red), swIAV group (green). a: *p* < 0.05; aa: *p* < 0.01; aaa: *p* < 0.001. SD0 (red arrow): PRRSV inoculation; SD8 (green arrow): swIAV inoculation.

The PRRSV genome was detected intermittently in nasal swab supernatants from PRRSV/swIAV and PRRSV groups without significant differences between both groups. Moreover, similar PRRSV genomic loads were quantified in sera from both PRRSV and PRRSV/swIAV groups throughout the experiment (Figure 2a). However, PRRSV genomic load was markedly affected in BALF from PRRSV/swIAV group from SD9 to SD15, with a sharp decrease at SD12, as compared to the PRRSV group (Figure 2b). Then, similar genomic loads were measured in BALF from both groups from SD21. Consistently, PRRSV infectious titers measured from SD9 to SD15 in BALF from the PRRSV/swIAV group were significantly lower than those measured in the PRRSV group at the same times (Table 3). At SD12, PRRSV infectious particles could not be detected in any samples from the PRRSV/swIAV group, whereas a PRRSV titer could be determined in 5/6 pigs from PRRSV group.

A slight delay in swIAV excretion was observed in nasal secretions from the PRRSV/swIAV group as compared to the swIAV group (Figure 2c). Indeed, only 2/6 and 3/6 pigs were found to excrete swIAV in the PRRSV/swIAV group at SD9 and SD10, respectively, compared with 4/6 and 5/6 in the swIAV group at these dates, respectively. At SD9, swIAV titration enabled detecting swIAV infectious particles in secretions from 2/6 pigs in the swIAV group, but not in samples from the PRRSV/swIAV group. However, from SD11, all swIAV-infected pigs excreted similar virus amounts. Moreover, in contrast to what was observed for PRRSV, similar swIAV genome loads were detected in BALF from both swIAV and PRRSV/swIAV groups from SD9 to SD15 (Figure 2d). Moreover, swIAV titers were equivalent in these samples. 

Thus, virological monitoring indicated that PRRSV replication in lungs was strongly disrupted following swIAV super-infection, whereas swIAV nasal shedding was only slightly delayed in PRRSV pre-infected pigs.

### 3.3. PRRSV Pre-Infection Attenuated Antiviral and Inflammatory Responses Induced by swIAV

Innate immune responses, i.e., antiviral (IFN-α), pro-inflammatory (IL-6), inflammatory (haptoglobin) and anti-inflammatory (IL-10) responses, were investigated in sera and/or BALF sampled during the time course of the study, and compared in the context of PRRSV and swIAV single infections versus the super-infection. 

**Figure 3:**
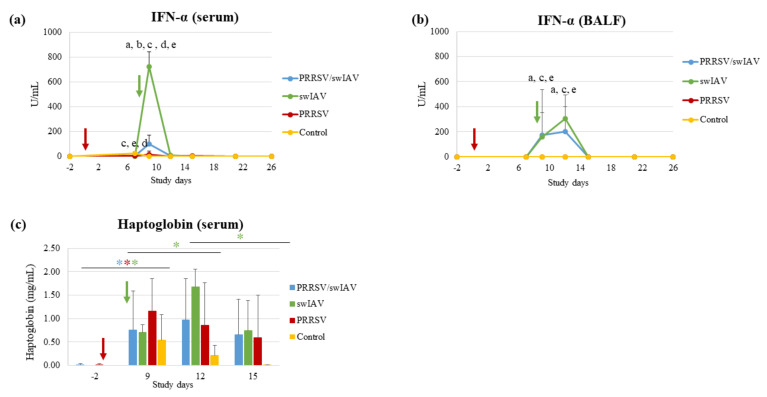
Quantification of IFN-α and haptoglobin. (**a**) Concentration of IFN-α in serum. (**b**) Concentration of IFN-α in BALF. (**c**) Concentration of haptoglobin in serum. All data are reported as the mean (±standard deviation) of results obtained from pigs (*n* = 6) in the PRRSV/swIAV (blue), PRRSV (red), swIAV (green) or Control (yellow) groups. Letters indicate that significant differences (with *p* < 0.05) were obtained between PRRSV/swIAV and (a) PRRSV and (b) swIAV and (c) Control groups, (d) and (e) significant difference between PRRSV and swIAV and Control groups (respectively).*: *p* < 0.05 comparing one time point to another within one group, * PRRSV/swIAV; * swIAV; * PRRSV. SD0 (red arrow): PRRSV inoculation; SD8 (green arrow): swIAV inoculation.

IFN-α was detected as soon as SD9 in serum and BALF from swIAV and PRRSV/swIAV groups (Figure 3a,b). However, at SD9, the serum concentration of IFN-α was markedly lower in the PRRSV/swIAV group, as compared to the swIAV group (*p* = 0.0005) (Figure 3a). In contrast, the mean IFN-α concentration in serum was significantly higher in the PRRSV/swIAV group than in the PRRSV group (*p* = 0.0003). In BALF, the IFN-α concentration increased from SD9 to SD12 for both the PRRSV/swIAV and swIAV groups, without significant differences (*p* > 0.05) (Figure 3b). However, as in serum, the IFN-α concentration in BALF was significantly higher in the PRRSV/swIAV group than in the PRRSV group (*p* = 0.0419 at SD9 and *p* = 0.0036 at SD12).

IL6 concentrations in sera and BALF from infected pigs were not quantifiable taking into account the limit of quantification given by the commercial kits. Nevertheless, by comparing IL-6 data in infected groups to the baseline level observed for the Control group, IL-6 induction was detected in sera from 4/6 pigs in the PRRSV/swIAV group and 6/6 pigs in the swIAV group at SD9. In BALF, IL-6 was detected in 3/6 and 5/6 pigs at SD9 and SD12, respectively, in each of the PRRSV/swIAV and swIAV groups, but not in the PRRSV group.

A significant increase in haptoglobin concentration in blood was observed from SD-2 to SD9 in all infected groups unlike in Control pigs (*p* = 0.0312 for the three groups between the two time points using paired comparisons) (Figure 3c). However, whereas a significant increase was also observed in the swIAV group from SD9 to SD12 (*p* = 0.0312), haptoglobin concentration remained as stable in the PRRSV/swIAV group as in the PRRSV group between these two time points. 

As for IL-6, IL-10 induction was analyzed qualitatively comparing to the baseline level of the Control group. Between SD9 and SD15, a slight induction of this anti-inflammatory cytokine was transiently detected in the BALF from 4/6 pigs in the PRRSV/swIAV group, 2/6 pigs in the swIAV group and 1/6 in the PRRSV group.

Thus, altogether, these results indicate that antiviral (IFN-α) and inflammatory (haptoglobin) responses were reduced in the PRRSV/swIAV group, as compared to the swIAV group.

### 3.4. PRRSV Pre-Infection Limited Granulocyte Influx in Blood and Lungs after swIAV Infection

Investigations were then extended to the effect the PRRSV/swIAV super-infection had on the dynamics of immune cells such as lymphocytes, macrophages and granulocytes in blood and lungs.

**Figure 4:**
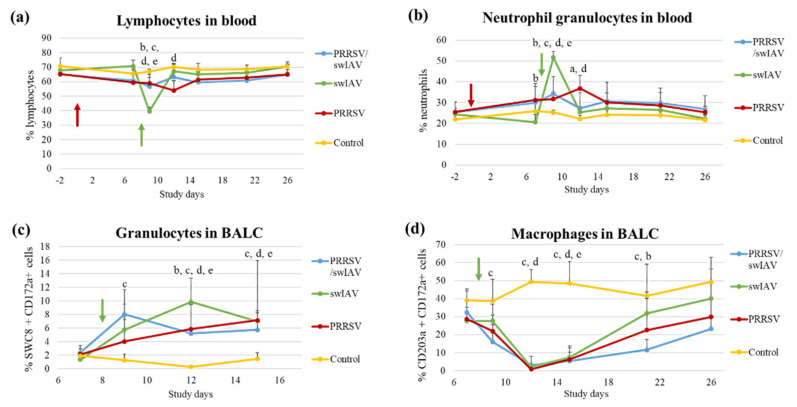
Immune cell proportions (percentages) in blood and among BALCs. (**a**) Blood lymphocytes. (**b**) Blood neutrophil granulocytes. (**c**) Granulocytes in BALC. (**d**) Macrophages in BALC. Data are reported as the mean (±standard deviation) percentages obtained from pigs (*n* = 6) in the PRRSV/swIAV (blue), PRRSV (red), swIAV (green) and Control (yellow) groups. Letters indicate that significant differences (with *p* < 0.05) were obtained between PRRSV/swIAV and (a) PRRSV and (b) swIAV and (c) Control groups, (d) and (e) significant difference between PRRSV and swIAV and Control groups (respectively). SD0 (red arrow): PRRSV inoculation; SD8 (green arrow): swIAV inoculation.

A drop in the percentage of blood lymphocytes was observed at SD9 in the swIAV group, as compared to the three other groups (*p* = 0.0006) (Figure 4a). At SD12, the proportion of lymphocytes stabilized in the swIAV group reached the same level as in other groups. In parallel, the percentage of blood neutrophil granulocytes increased in the swIAV group at SD9, reaching a proportion that was significantly higher than that evaluated in the PRRSV/swIAV group (*p* = 0.0013) (Figure 4b). 

At the lung level, the influx of granulocytes within BALCs at SD12 was significantly higher in the swIAV group than in the PRRSV/swIAV group (*p* = 0.0475) (Figure 4c). In the meantime, a strong decrease in the proportions of collected lung macrophages was observed in the three infected groups as compared to the Control group (Figure 4d). However, the recovery in this cell population from SD15 was slower in the PRRSV/swIAV group, leading to significantly lower macrophage percentages at SD21 as compared to the swIAV group (*p* = 0.0333). 

Thus, these findings mainly revealed that the influx of granulocytes was limited at systemic and pulmonary levels in the PRRSV/swIAV group, as compared to the swIAV group.

### 3.5. PRRSV Pre-Infection Enhanced the Anti-swIAV Humoral Immune Response in Lungs

Humoral immune responses to PRRSV and swIAV infections were evaluated through the detection of antibodies specific to each virus.

No difference in anti-PRRSV IgG antibody levels was observed in sera from PRRSV and PRRSV/swIAV groups in the time course of the experiment (Figure S3a). Similarly, no differences in anti-PRRSV IgG and IgA antibody levels were observed in BALF obtained from PRRSV and PRRSV/swIAV groups (Figure S3b,c). Moreover, no PRRSV-specific neutralizing antibodies were detected in sera from PRRSV and PRRSV/swIAV groups at SD26.

**Figure 5:**
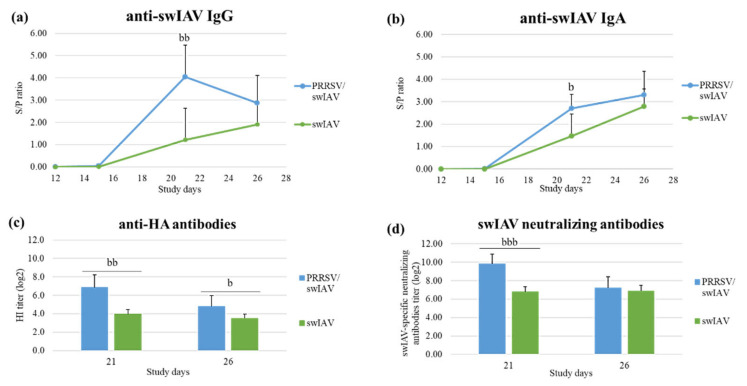
Anti-swIAV antibodies in BALF from infected groups. (**a**) IgG and (**b**) IgA (NP protein) levels. (**c**) Anti-HA antibody and (**d**) swIAV-neutralizing antibody titers. Data are reported as means (±standard deviation) of results obtained from pigs (*n* = 6) in the PRRSV/swIAV (blue) and swIAV (green) groups. b: *p* < 0.05; bb: *p* < 0.01; bbb: *p* < 0.001. SD0: PRRSV inoculation; SD8: swIAV inoculation.

Levels in anti-swIAV IgG, anti-hemagglutinin and swIAV-specific neutralizing antibodies were similar in sera from swIAV and PRRSV/swIAV groups at all collection times (Figure S4). In contrast, anti-swIAV IgG and IgA levels were significantly higher in BALF obtained at SD21 in the PRRSV/swIAV group as compared to the swIAV group (*p* = 0.0032 for anti-swIAV IgG, and *p* = 0.0289 for anti-swIAV IgA) (Figure 5a,b). In addition, the mean hemagglutination inhibition titer was higher in the PRRSV/swIAV group than in the swIAV group at SD21 (*p* = 0.0011) and SD26 (*p* = 0.0329). Consistently, mean titers in swIAV-neutralizing antibodies were higher in the PRRSV/swIAV group than in the swIAV group at SD21 (*p* < 0.0001) (Figure 5c,d).

IL-4 might be a marker of the stimulation of the humoral response; therefore, we also attempted to measure the IL-4 concentration in BALF, but IL-4 was not detected in the available samples. Altogether, these analyses demonstrated a stronger humoral response against swIAV in the lungs of pigs from the PRRSV/swIAV group as compared to the swIAV group.

### 3.6. The Cell-Mediated Immune Response Specific to PRRSV was Induced Faster in Super-Infected Pigs

The cell-mediated immune (CMI) responses specific to each virus were evaluated using ELISPOT IFN-γ assays on PBMC.

**Figure 6:**
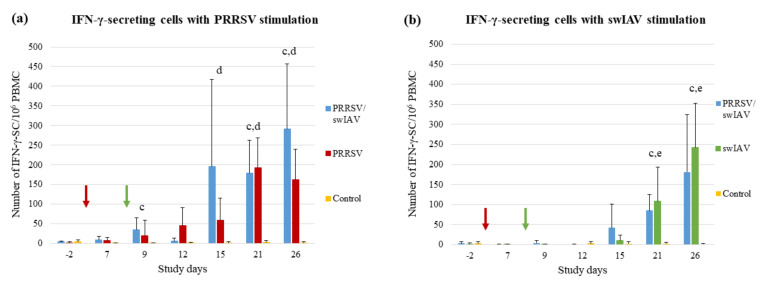
Cell-mediated immune response. Number of IFN-γ-secreting cells per million PBMCs after (**a**) PRRSV stimulation, or (**b**) swIAV stimulation. Data are reported as the means (±standard deviation) of results obtained from pigs (*n* = 6) in the PRRSV/swIAV (blue), PRRSV (red), swIAV (green) or Control (yellow) groups. Letters indicate that significant differences (with *p* < 0.05) were obtained between PRRSV/swIAV and (a) PRRSV and (b) swIAV and (c) Control groups and (d) and (e) between PRRSV and swIAV and the Control groups (respectively), SD0 (red arrow): PRRSV inoculation; SD8 (red arrow): swIAV inoculation.

The follow-up of the PRRSV-specific immunity indicated a fast induction of the CMI response in the PRRSV/swIAV group, with a mean number of IFN-γ-secreting cells (IFN-γ-SC) significantly higher than that measured in the Control group at SD9 (*p* = 0.0452) (Figure 6a). Conversely, no difference was observed between PRRSV and Control groups at that time. Moreover, 3/6 pigs from the PRRSV/swIAV group showed a strong PRRSV-specific CMI response at SD15, with a number of IFN-γ-SC higher than 250 per million of PBMCs. In contrast, none of the pigs from the PRRSV group exceeded 200 IFN-γ-SC/10^6^ PBMCs at the same time point. Then, at SD21 and SD26, both PRRSV/swIAV and PRRSV groups exhibited significantly higher CMI responses than the Control group.

Following swIAV stimulation, no significant difference in the number of IFN-γ-SC was observed between PRRSV/swIAV and swIAV groups, whatever the follow-up time point. At SD21 and SD26, the swIAV-specific CMI responses from both PRRSV/swIAV and swIAV groups were significantly different from the Control group (Figure 6b).

IFN-γ measurements in BALF were carried out using ELISAs because the induction of this cytokine in lungs could have revealed differences in CMI initiation; however, IFN-γ was not detected in any of the samples from infected pigs at the time they were taken.

Overall, it appeared that the CMI response specific to PRRSV was induced faster in super-infected pigs, whereas that specific to swIAV was not modified, as compared to PRRSV or swIAV single-infected groups.

### 3.7. Correlation Analyses

To explore the links between the clinical, virological and immunological parameters we monitored in this study, we performed PCAs and correlation tests using the different collected data.

**Figure 7:**
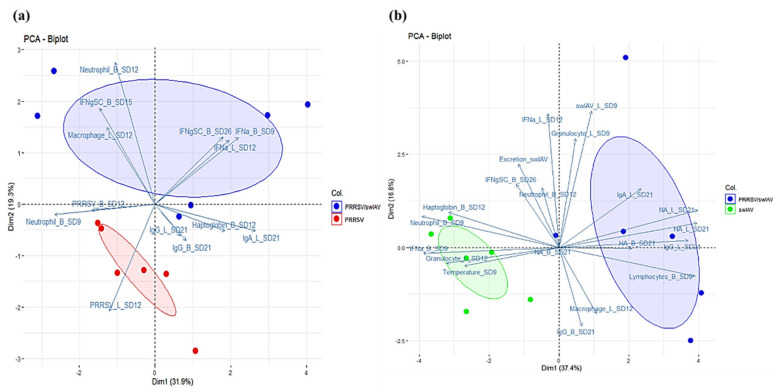
Principal component analysis (PCA) of the experimental variables of co-infected and single-infected pigs. (**a**) PCA biplot of PRRSV/swIAV and PRRSV pigs (13 factors). (**b**) PCA biplot of PRRSV/swIAV and swIAV pigs (20 factors). L: lungs; B: blood; SD: study day; PRRSV: PRRSV genomic load; swIAV: swIAV genomic load; HA: anti-HA antibodies; NA: swIAV-neutralizing antibodies. IFNgSC: CMI. Dim 1 is the axis representing the highest percentage of variance and Dim 2 represents the second axis. Each color dot (PRRSV/swIAV: blue, PRRSV: red or swIAV: green) represents the projection on the PCA of each pig. The ellipses depict the spread of the PCA data for PRRSV/swIAV, PRRSV or swIAV groups.

Correlations related to PRRSV infection included data from PRRSV/swIAV and PRRSV groups. PCA revealed that IFN-α concentrations in BALF at SD12 were positively correlated with PRRSV-specific CMI responses (IFN-γ-SC) at SD26. Moreover, IFN-α concentrations in serum at SD9 and in BALF at SD12 were negatively correlated with PRRSV replication in BALF at SD12 (Figure 7a). These results were confirmed by a Spearman correlation analysis (Figure S1). 

Correlations related to swIAV infection comprised data from PRRSV/swIAV and swIAV groups. PCA revealed that rectal temperature at SD9, IFN-α level at SD9, haptoglobin level at SD12 and percentage of granulocytes within BALC at SD12 were positively correlated between them, what was confirmed by a Spearman correlation analysis (Figure S2). Moreover, the humoral immune response measured in BALF at SD21 (swIAV-specific neutralizing, anti-hemagglutinin, and anti-swIAV IgG and IgA antibodies) was found to be anti-correlated with the above parameters (clinical signs, inflammatory and antiviral responses) (Figure 7b). Finally, rectal temperature or haptoglobin concentration were not related to swIAV multiplication, whether measured in nasal secretions or in lungs. 

Therefore, altogether these data suggested that IFN-α response had probably played a primary role in the interference between both infections, it is noteworthy that the dispersions of data showed in both Figure 7a,b (represented by the blue, red and green ellipses) are much higher for the super-infected group compared to the single-infected groups, suggesting that super-infection leads to more heterogeneous responses than PRRSV or swIAV single-infections.

## 4. Discussion

In this study, we investigated the possible interference between PRRSV-1 and swIAV (H1N2) infections in an experimental model based on successive inoculations eight days apart. This model mimics as best as possible a situation most likely to be encountered in farms, taking into account the long-lasting and acute profiles of PRRSV and swIAV infections, respectively. Our laboratory has gained considerable experience of each single PRRSV and swIAV infection model [3,10,28], which made it possible to interpret the data obtained here in this super-infection context.

The first objective was to investigate the clinical and immuno-virological outcomes of a PRRSV-1 infection on a subsequent swIAV (H1N2) infection. In a previous study, where we inoculated H1N2 swIAV intratracheally to 9-week-old SPF pigs, reductions in food consumption and weight gain were evidenced during the first four days post-inoculation, before recovering [28]. In the present study, individual weighing was not performed daily; thus, we did not observe such an early impact of the swIAV infection. Nevertheless, what can be noticed is that any difference in growth performance was observed between the infected groups in the second and third weeks post-inoculation with swIAV. This suggests that even if swIAV infection had impacted food consumption and weight gain in the first days, PRRSV pre-infection had not exacerbated this swIAV infection outcome, and/or the PRRSV/swIAV group recovered at least as quickly as the swIAV group. In any case, hyperthermia and respiratory signs were observed in the swIAV group, as expected. In contrast, fewer clinical signs were observed in the super-infected group, suggesting an attenuation of H1N2 swIAV infection outcomes in the PRRSV-1 pre-infected pigs. Knowing that pig lungs are largely destabilized by PRRSV infection eight days post-inoculation [10], one might have expected an exacerbation of swIAV clinical signs in the PRRSV/swIAV group, rather than an attenuation. In addition, our results were consistent with most of the few experimental studies that previously investigated PRRSV-1/swIAV co-infection, because most of them did not evidence an exacerbation of the influenza disease [21,23,24]. Indeed, whereas a first study of Van Reeth et al. suggested an enhancement of clinical disease in some conventional pigs inoculated with PRRSV-1 and H1N1 swIAV three days apart [20], large variations in the clinical responses were observed between individual pigs, which was confirmed in further experiments where inoculations were performed either three or seven days apart [21]. However, in colostrum-deprived caesarian-derived (CDCD) piglets inoculated in seven-day intervals, the differences with the single virus infections were negligible [21]. The same observation, i.e., no significant impact of PRRSV infection on subsequent swIAV infection, was reported when SPF piglets were inoculated with PRRSV-1 and H3N2 swIAV one week apart [23], or when conventional pigs were simultaneously inoculated with PRRSV-1 and H1N1 swIAV [24]. Interestingly, the dual infection was largely subclinical in conventional pigs inoculated with PRRSV-1 and H1N1 swIAV with a 14-day interval [21], in line with the attenuation of swIAV clinical signs we observed here in SPF pigs inoculated with PRRSV-1 and H1N2 swIAV eight days apart. Altogether, whereas the diversity of the experimental protocols could make it difficult to compare them, it seems that both the sanitary status of pigs (conventional, SPF or CDCD) as well as the time interval between inoculations (0, 3, 7, 8 or 14 days) can affect the clinical outcome of PRRSV-1/swIAV super-infection, whereas impact of the age of the animals (3 to 12 week old), the inoculation routes (nebulization, nasal, tracheal) or the swIAV subtype (H1N1, H3N2, H1N2) cannot be excluded at this stage. Although the inoculation route does not influence the outcomes of a single PRRSV infection [35], it is known that the severity of influenza disease is dependent upon swIAV delivery, as observed in our lab (unpublished results) and reported by others [36]. Tracheal inoculation is a technique which enables the reproduction of clinical outcomes observed in the field, the induction of marked clinical signs, as well as pulmonary inflammation, the observation of their attenuation in pigs pre-infected with PRRSV-1 is all the more relevant. Further comparative experimental assays using nebulization or shedder pigs as a more natural inoculation route for swIAV could be considered to confirm the results we obtained here. 

The swIAV subtype may also play a role in the outcomes of the super-infection. Indeed, we have previously shown that the H1N2 swIAV strain used in this study was a little more pathogenic than an H1N1 swIAV strain, but outcomes of H1N2 infection in pigs infected with Mhp three weeks before the swIAV inoculation were not exacerbated, in contrast to outcomes of H1N1 infection in such a context of super-infection [37].

Correlation analyses suggested that the mitigation of clinical signs we have observed here in super-infected animals was positively correlated with the attenuation of the inflammatory response. Indeed, we observed a peak of haptoglobin in the swIAV group at 4 days post-swIAV infection as previously shown [28,38], but not in the PRRSV/swIAV group. Consistently, an influx of granulocytes was observed in the lungs of pigs from swIAV group as expected [28], whereas it was more limited in super-infected group. Interestingly, PCAs also indicated that the attenuation of clinical outcomes was not related to swIAV multiplication, because similar swIAV genomic loads were measured in the lungs of swIAV and PRRSV/swIAV groups. The absence of effect of PRRSV pre-infection on swIAV multiplication in lungs may result from differences in cellular targeting between the two viruses. In support of this hypothesis, it can be noted that in a co-infection study using two viruses replicating both in the lower respiratory epithelium, namely, porcine respiratory coronavirus (PRCV) and swIAV, PRCV infection strongly decreased swIAV replication [39]. In contrast, another study on PRRSV/PRCV co-infection showed no effect of PRRSV on PRCV replication and shedding [20].

However, clinical outcomes and inflammatory systemic response were found to be positively correlated with IFN-α levels. swIAV infection is known to be a high inducer of type-I IFN [40], whereas PRRSV is deemed to block such an induction [41]. Type-I IFN promotes both innate anti-viral defenses and adaptive immunity, while also bearing a deleterious role by inducing inflammatory responses which can lead to clinical manifestations [42]. Thus, the links that were evidenced here between inflammatory and IFN responses are in accordance with results previously reported by others in the context of swIAV single infections [28,43,44]. Nevertheless, the IFN-α level was significantly reduced in super-infected pigs, as compared to swIAV-infected pigs, suggesting that PRRSV pre-infection interfered with IFN-α induction. This result is consistent with an in vitro study showing that macrophages infected with PRRSV and then with a swine alphacoronavirus, which is a good IFN-α inducer as swIAV, failed to produce the antiviral cytokine [45]. Interestingly, another in vitro investigation indicated that PRRSV could interfere with the JAK-STAT signaling pathway in newborn porcine tracheal (NPTr) epithelial cells, even without entering these cells, leading to a sub-expression of IFN-stimulated genes after infection with swIAV [46].

Similar levels of antibodies directed against H1N2 swIAV were measured in sera from swIAV and PRRSV/swIAV groups, as reported by others when comparing humoral responses at the systemic level in H1N1-infected and PRRSV/H1N1 co-infected pigs [24]. Conversely, we detected higher levels of anti-swIAV antibodies in BALF from the PRRSV/swIAV group, as compared to the swIAV group, at SD21. PRRSV infection induces polyclonal hypergammaglobulinemia [47]; therefore, this phenomenon may have played a role in the high production of anti-swIAV antibodies in BALF from the super-infected group. However, no correlation between the levels of anti-swIAV antibodies in BALF and swIAV multiplication was observed, suggesting that this huge humoral response had no impact on swIAV clearance. In parallel, a slight induction of IL-10 was measured in the lungs of more pigs from the PRRSV/swIAV group, than from the single-infected group, as already observed by others after PRRSV/swIAV co-infection [48]. However, due to the low sensitivity of IL-10 detection in BALF by ELISA, additional comparative analyses of IL-10 using other methods such as gene expression quantification in BALCs of infected groups [10] should be considered to confirm its higher induction in the lungs of super-infected pigs.

The second objective of this study was to investigate the effect of swIAV infection on the course of an ongoing PRRSV infection. Interestingly, a transient but strong decrease in PRRSV genomic load was observed in the lungs of PRRSV/swIAV superinfected pigs, as compared to PRRSV single-infected pigs, in the few days after swIAV inoculation, consistent with other studies regarding PRRSV/swIAV coinfection in pigs [49]. Host target cells for PRRSV are alveolar macrophages [8], whereas swIAV mainly infects epithelial cells of upper and lower respiratory tracts [13]; therefore, this viral interference should depend on indirect mechanisms. Knowing that PRRSV is very sensitive to IFN-α [45], and as supported by correlation analyses, the impact that swIAV infection had on PRRSV multiplication was probably linked to the induction of IFN-α in the lungs of PRRSV/swIAV co-infected pigs. As mentioned above, IFN-α levels were strongly reduced as compared to those measured in swIAV-infected pigs, but still higher than usually measured after PRRSV infection. These results are completely in line with a previous study showing that the induction of IFN-α through a non-replicating adenovirus (Ad5-IFN-α) inhibited the replication of a PRRSV-2 strain in pigs inoculated one-day later [50]. In the same way, we showed, in a recent study, that a concomitant swIAV infection can temporarily inhibit the replication of a PRRSV-1-modified live vaccine, probably through IFN-α induction [51]. In contrast, the fact that the IFN-α concentration was low in blood could explain why the PRRSV genomic load was not affected at the systemic level. Nevertheless, in addition to IFN-α, the role of other cytokines in decreasing PRRSV multiplication in lungs, such as tumor necrosis factor alpha (TNF-α), cannot be excluded. Even though we were not able to detect any TNF-α in the lungs after H1N2 inoculation in a previous study [28], others have reported that swIAV infection could lead to an increase in TNF-α concentration in the lungs [48]. If this happened, TNF-α could have played a role because it was shown to have an antiviral effect on PRRSV [52,53]. 

No difference between PRRSV and PRRSV/swIAV groups was observed regarding the humoral response against PRRSV, as also previously described [24]. However, we observed that the number of PRRSV-specific IFN-γ-SCs increased faster in the blood of pigs from the PRRSV/swIAV group as compared to the PRRSV group. A correlation was found between the IFN-α level and the PRRSV-specific CMI response; therefore, it could be hypothesized that IFN-α induction linked to swIAV infection might have stimulated the cellular response against PRRSV. This is supported by another study that also showed a significant increase in the number of PRRSV-specific IFN-γ-SCs in pigs injected with an Ad5-pIFN-α at the time of PRRSV inoculation, as compared to single-infected pigs [54]. Beyond innate anti-viral defenses, IFN-α can also activate the CMI response by stimulating the dendritic cells that trigger antigen-specific T cell proliferation [55].

In conclusion, in our experimental conditions, this study demonstrated an interplay between PRRSV and swIAV infections, with a two-way interference that most likely involved IFN-α in different roles. On the one hand, reductions in IFN-α levels in superinfected pigs, as compared to swIAV-infected pigs, would have contributed to the attenuation of influenza-like illness through the impairment of inflammatory response. On the other hand, increases in IFN-α in super-infected pigs, as compared to PRRSV single-infected pigs, would have counteracted PRRSV multiplication and stimulated the induction of CMI specific to PRRSV. However, other cytokines could have played a role in viral interference, which deserves further investigation. Additionally, as underlined previously, the results we obtained here might have been different when using other viral subtypes or another delay between inoculations or pigs with a different health status. Further studies evaluating the role of each of these parameters in the outcomes of super-infection are thus still needed.

However, even if such an experimental super-infection model may not still represent the complexity of the situation encountered in the field [2], and because other factors so far unknown could also play a role in the variations noticed in the responses between individual pigs, this study provides new knowledge about interactions between two respiratory RNA viruses of importance in pig production. It suggests that an antagonistic, but not a synergistic or additive, effect may result from a co-infection, probably depending on event chronology, as also recently evidenced in vitro in a study dedicated to dual infection with swIAV (H3N2) and porcine respiratory coronavirus [56]. Although further investigations are needed to more deeply decipher the complex interplays between PRRSV and swIAV infections, these findings provide new insight into PRDC regulation in pigs [2]. They should be relevant as well for the comprehensive understanding of respiratory virus co-infections in other species, such as within bovine respiratory disease in cattle [57], or in humans where co-infections may occur with influenza A virus and other viruses [58], among which some exhibit an ability to interfere with the IFN pathway.

## Figures and Tables

**Table 1:** Experimental design.

Group	PRRSV Inoculation	swIAV Inoculation	Necropsy
PRRSV	SD0	-	SD27
swIAV	-	SD8	SD29
PRRSV/swIAV	SD0	SD8	SD28
Control	-	-	SD27 and SD28

SD: Study day; -: mock-inoculation.

**Table 2:** List of variables included in principal component analyses.

Variables	Study Day	Sample
Rectal temperature	SD9	-
Haptoglobin	SD12	Serum
PRRSV genomic load	SD12	Serum and BALF
swIAV genomic load	SD9	BALF
Duration of swIAV nasal excretion	From SD9 to SD26	Nasal swab supernatants
IFN-α	SD9 and SD12	Serum and BALF
Neutrophils	SD9 and SD12	Blood
Granulocytes	SD9 and SD12	BALC
Macrophages	SD12	BALC
Lymphocytes	SD9	Blood
PRRSV-specific humoral response: anti-IgG, anti-IgA	SD21	Serum and BALF
swIAV-specific humoral response: anti-IgG, anti-IgA, anti-HA and neutralizing antibodies	SD21	Serum and BALF
PRRSV specific IFN-γ-SC	SD15 and SD26	Blood
swIAV specific IFN-γ-SC	SD26	Blood

**Table 3:** Mean PRRSV infectious titers (log10 TCID_50_/mL) (±standard deviation) measured from SD7 to SD21 in BALF from PRRSV/swIAV and PRRSV groups.

Group	SD7	SD9	SD12	SD15	SD21
PRRSV/swIAV	2.87 ± 0.87 (6/6)	3.47 ± 0.48 * (6/6)	0 * (0/6)	2.90 ± 0.45 * (6/6)	1.70 ± 1.43 (4/6)
PRRSV	2.26 ± 1.27 (5/6)	4.50 ± 0.47 (6/6)	2.20 ± 1.15 (5/6)	3.50 ± 0.47 (6/6)	0.33 ± 0.82 (1/6)

TCID_50_/mL: 50% tissue culture infectious dose per milliliter; SD: study day; (*n*/6): number of pigs with infectious particles titrated. Zero has been assigned when PRRSV infectious particles could not be titrated in samples. * Significantly different from the PRRSV group.

## Data Availability

Not applicable.

## Supplementary Materials

The following are available online at https://www.mdpi.com/article/10.3390/v13112169/s1. Figure S1: Correlation analysis between variables obtained in the PRRSV/swIAV and PRRSV groups. Spearman’s correlation coefficients between haptoglobin concentration in blood at SD12, PRRSV genomic load in the blood and lungs at SD12, concentration of IFN-α in blood at SD9 and in the lungs at SD12, the percentage of neutrophils in the blood at SD9, the percentage of granulocytes in the lungs at SD12, the percentage of macrophages in the lungs at SD12, the level of anti-PRRSV IgG in blood and lungs at SD21, the level of anti-PRRSV IgA in the lungs at SD21, and the number of IFN-γ-SCs in the blood at SD15 and SD26. Grey cells indicate that the correlation was significant at *p* < 0.05. B: blood, L: lung, SD: study day. Figure S2: Correlation analysis between parameters obtained in the PRRSV/swIAV and swIAV pigs. Spearman’s correlation coefficients between rectal temperature at SD9, the concentration of haptoglobin in blood at SD12, the swIAV genomic load in lungs at SD9, the duration of swIAV excretion (in days), the concentration of IFN-α in the blood at SD9 and in the lungs at SD12, the percentage of neutrophils in the blood at SD9 and SD12, the percentage of granulocytes in the lungs at SD9 and SD12, the percentage of macrophages in the lungs at SD12, the percentage of lymphocytes in the blood at SD9, the levels of swIAV-specific IgG, IgA, anti-HA, neutralizing antibodies in the lungs and in serum at SD21, the number of IFN-γ-SC per million PBMC (in blood) at SD26. Grey cells indicate that the correlation is significant at *p* < 0.05. B: blood, L: lungs, SD: study days. Figure S3: Detection of anti-PRRSV antibodies in serum and BALF from infected groups. (a) Anti-PRRSV IgG in serum, (b) Anti-PRRSV IgG in BALF, (c) Anti-PRRSV IgA in BALF. Data are reported as the means (±standard deviation) of results obtained from pigs (*n* = 6) in the PRRSV/swIAV (blue) and PRRSV (red) groups. SD0 (red arrow): PRRSV inoculation; SD8 (green arrow): swIAV inoculation. Figure S4: Anti-swIAV antibodies in serum from infected groups. (a) Detection of anti swIAV IgG in serum; (b) Titration of anti-HA antibodies in serum; (c) Titration of swIAV-specific neutralizing antibodies in serum. Data are reported as the means (±standard deviation) of results obtained from pigs (*n* = 6) in the PRRSV/swIAV (blue) and swIAV (green) groups. SD0 (red arrow): PRRSV inoculation; SD8 (green arrow): swIAV inoculation.
