# Supplementary figures and images for: Successive Inoculations of Pigs with Porcine Reproductive and Respiratory Syndrome Virus 1 (PRRSV-1) and Swine H1N2 Influenza Virus Suggest a Mutual Interference between the Two Viral Infections
Source: Viruses. 2021 Oct 27;13(11):2169. doi: 10.3390/v13112169 (PMC8625072; doi:10.3390/v13112169)

**(a) anti-PRRSV IgG (serum)**

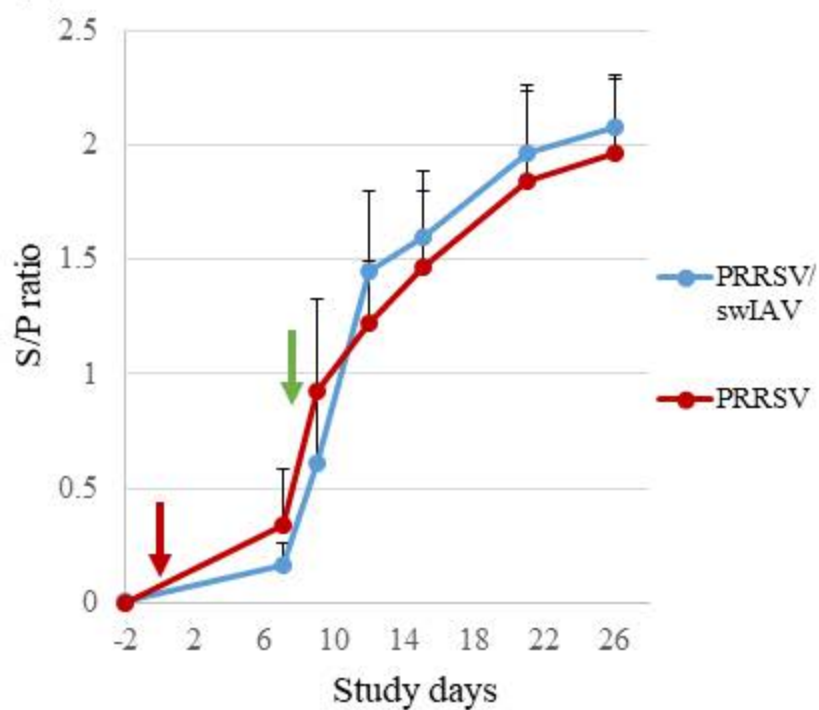

**(b) anti-PRRSV IgG (BALF)**

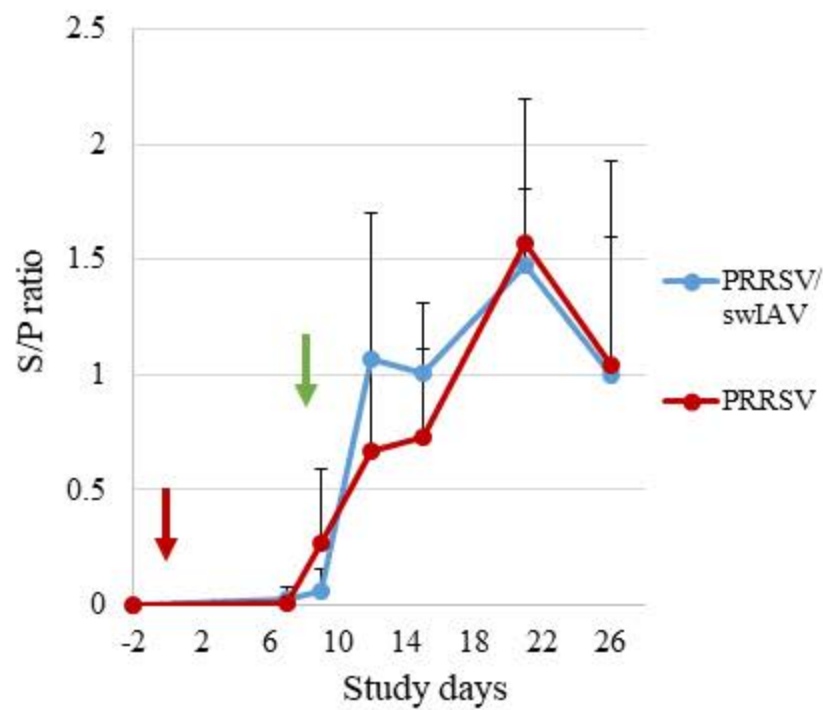

**(c) Anti-PRRSV IgA (BALF)**

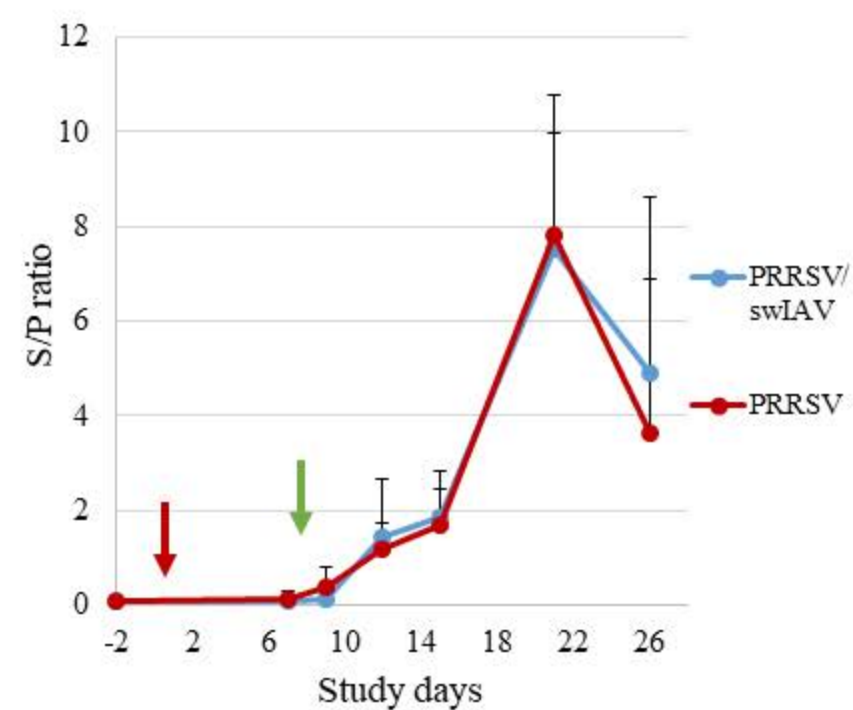

Supplement: Supplementary file 1 [file viruses-13-02169-s001.zip › Figure S3.pdf]

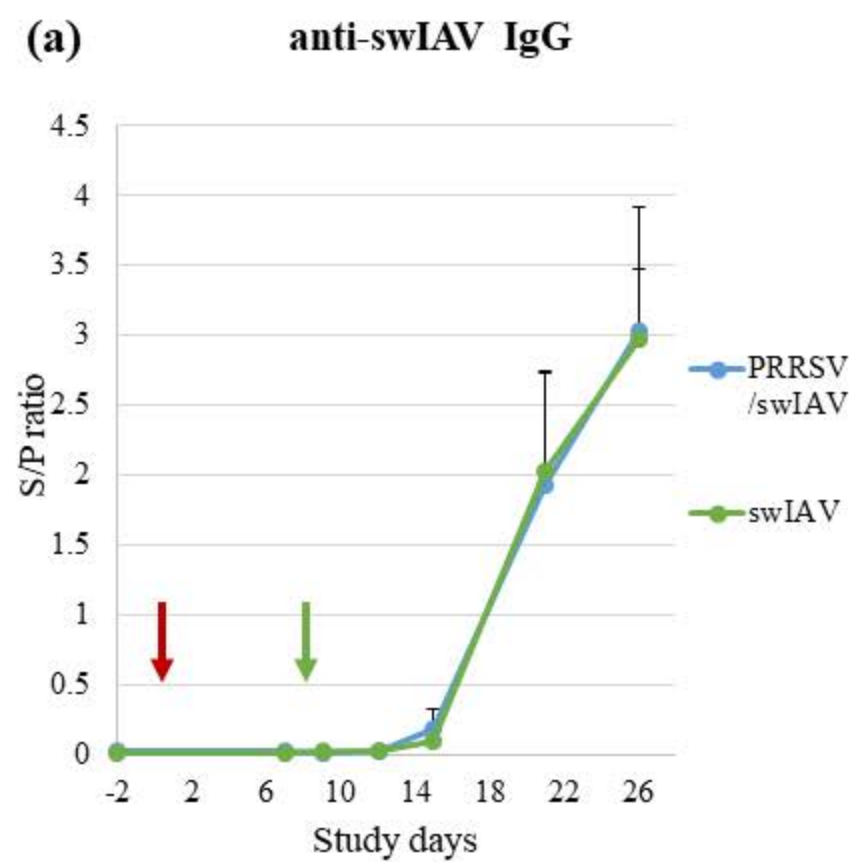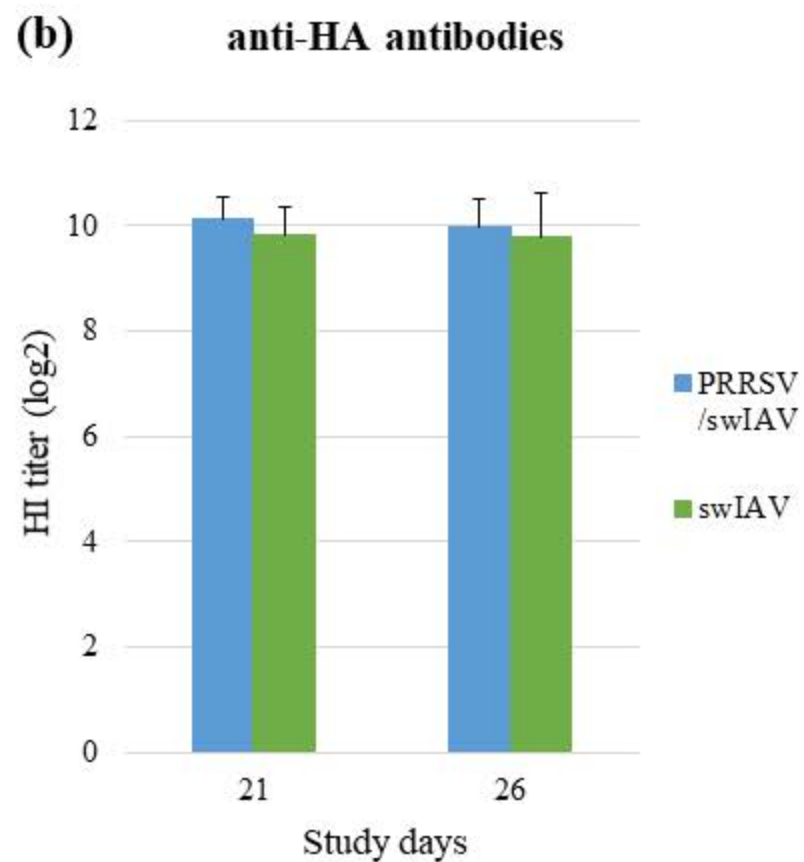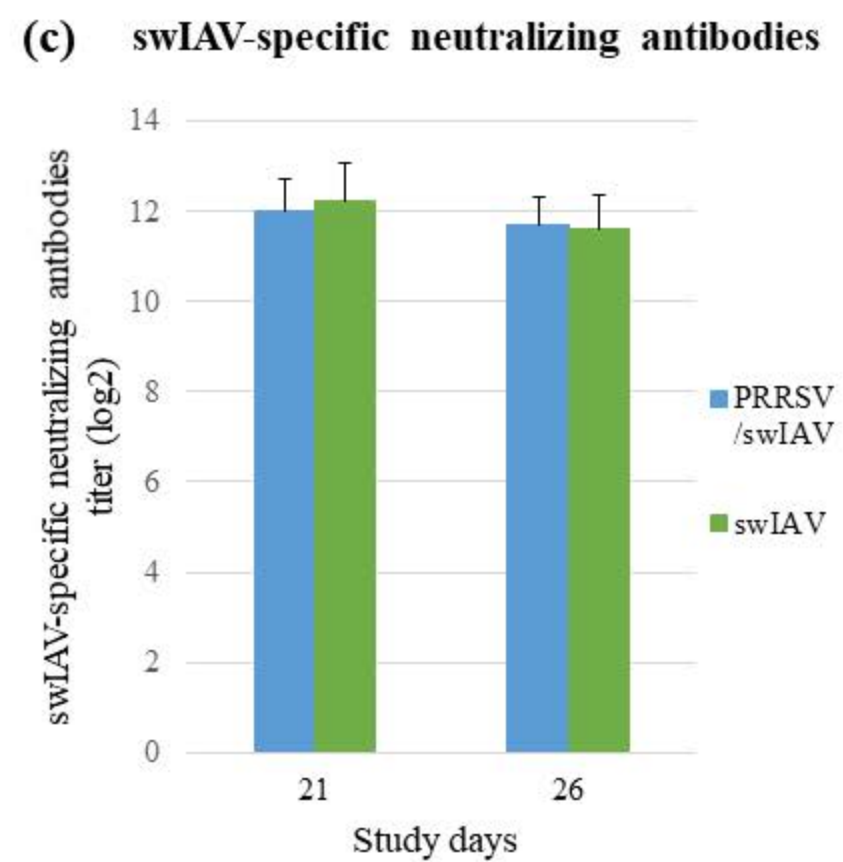

Supplement: Supplementary file 1 [file viruses-13-02169-s001.zip › Figure S4.pdf]
